# Piezo2 Knockdown Inhibits Noxious Mechanical Stimulation and NGF-Induced Sensitization in A-Delta Bone Afferent Neurons

**DOI:** 10.3389/fphys.2021.644929

**Published:** 2021-07-15

**Authors:** Sara Nencini, Michael Morgan, Jenny Thai, Andrew I. Jobling, Stuart B. Mazzone, Jason J. Ivanusic

**Affiliations:** Department of Anatomy and Physiology, University of Melbourne, Melbourne, VIC, Australia

**Keywords:** bone, bone pain, skeletal, skeletal pain, Piezo2, NGF, mechano-nociception

## Abstract

Piezo2 is a mechanically gated ion-channel that has a well-defined role in innocuous mechanical sensitivity, but recently has also been suggested to play a role in mechanically induced pain. Here we have explored a role for Piezo2 in mechanically evoked bone nociception in Sprague Dawley rats. We have used an *in vivo* electrophysiological bone-nerve preparation to record the activity of single Aδ bone afferent neurons in response to noxious mechanical stimulation, after Piezo2 knockdown in the dorsal root ganglia with intrathecal injections of Piezo2 antisense oligodeoxynucleotides, or in control animals that received mismatch oligodeoxynucleotides. There were no differences in the number of Aδ bone afferent neurons responding to the mechanical stimulus, or their threshold for mechanical activation, in Piezo2 knockdown animals compared to mismatch control animals. However, bone afferent neurons in Piezo2 knockdown animals had reduced discharge frequencies and took longer to recover from stimulus-evoked fatigue than those in mismatch control animals. Piezo2 knockdown also prevented nerve growth factor (NGF)-induced sensitization of bone afferent neurons, and retrograde labeled bone afferent neurons that expressed Piezo2 co-expressed TrkA, the high affinity receptor for NGF. Our findings demonstrate that Piezo2 contributes to the response of bone afferent neurons to noxious mechanical stimulation, and plays a role in processes that sensitize them to mechanical stimulation.

## Introduction

Pain associated with bone marrow edema syndromes, osteomyelitis, osteoarthritis, fractures, and bone cancer puts a significant burden (both in terms of quality of life and cost) on individuals, society, and health care systems worldwide. A feature common to almost all conditions that produce bone pain is the presence of a mechanical disturbance of the bone, and this is the most likely trigger for pain (Lemperg and Arnoldi, 1978; Arnoldi et al., 1980; Haegerstam, 2001; Kidd et al., 2004; Urch, 2004; Starr et al., 2008; Bove et al., 2009; Mantyh, 2014). Importantly, the identity of molecules that transduce noxious mechanical stimuli in bone, and/or influence the mechanical sensitivity of bone nociceptors, has not yet been established. This has limited our understanding of how nociceptors code information about mechanical disturbances in bone, and has hampered attempts to target specific therapies to treat mechanically induced bone pain.

Piezo2 is a mechanically gated ion-channel that has received significant attention, in part because of its remarkable structure (Coste et al., 2010, 2012; Wang et al., 2019). There is strong evidence that Piezo2 is the transducer for low-threshold mechanical stimuli in Merkel cells (Ikeda et al., 2014; Ikeda and Gu, 2014; Ranade et al., 2014; Woo et al., 2014) and proprioceptors (Woo et al., 2015; Florez-Paz et al., 2016). However, more recent evidence suggests Piezo2 might also be involved in the transduction of noxious mechanical stimuli (Dubin et al., 2012; Eijkelkamp et al., 2013; Singhmar et al., 2016; Murthy et al., 2018; Szczot et al., 2018). Mechanically activated Piezo2 currents are enhanced by the algesic peptide bradykinin that drives mechanical hypersensitivity associated with inflammation (Dubin et al., 2012). Piezo2 knockdown in dorsal root ganglia (DRG) inhibits inflammation-induced mechanical but not thermal hyperalgesia in mouse skin (Singhmar et al., 2016) and attenuates viscero-motor pain reflexes in response to noxious and innocuous colorectal distension in rats (Yang et al., 2016b). Furthermore, Piezo2 knockout does not prevent cutaneous nociceptors from transducing mechanical stimuli but it does reduce the sensitivity of mechano-nociceptors in the skin-nerve preparation (Ranade et al., 2014; Murthy et al., 2018). Previous studies have also identified interactions of Piezo2 with other proteins, for example nerve growth factor (NGF), that together contribute to the mechanical sensitivity of peripheral sensory neurons (Qi et al., 2015; Prato et al., 2017).

In a previous study, we have shown that a substantial proportion of peripheral sensory neurons that innervate bone (bone afferent neurons) express Piezo2, that the majority of myelinated (neurofilament rich) bone afferent neurons express Piezo2, and that Piezo2 expression is rare in unmyelinated (neurofilament poor) bone afferent neurons (Nencini and Ivanusic, 2017). However, there have been no attempts to identify a physiological role for Piezo2 signaling in bone pain.

We have recently developed a novel *in vivo* electrophysiological bone-nerve preparation that allows us to record, for the first time, the activity of different populations of bone afferent neurons in response to noxious mechanical stimulation (Nencini and Ivanusic, 2017; Nencini et al., 2017, 2018, 2019; Morgan et al., 2019; Morgan et al., 2020). In the present study, we use this approach to determine how Piezo2 knockdown affects the ability of bone afferent neurons to respond to noxious mechanical stimulation applied to the bone. We also tested whether Piezo2 knockdown affects NGF-induced sensitization of bone afferent neurons to mechanical stimulation. Our findings provide clear evidence for a role of Piezo2 in bone nociception.

## Materials and Methods

### Ethical Approval and Animal Care

Male Sprague-Dawley rats weighing between 200 and 250 g were used in this study. Animals were sourced from the Biomedical Sciences Animal Facility at the University of Melbourne. Animals were housed in pairs or groups of four, in a 12/12 h light/dark cycle and were provided with food and water *ad libitum*. All experiments conformed to the Australian National Health and Medical Research Council code of practice for the use of animals in research and were approved by the University of Melbourne Animal Experimentation Ethics Committee.

### Piezo2 Knockdown Using Antisense Oligodeoxynucleotides

**FIGURE 1:**
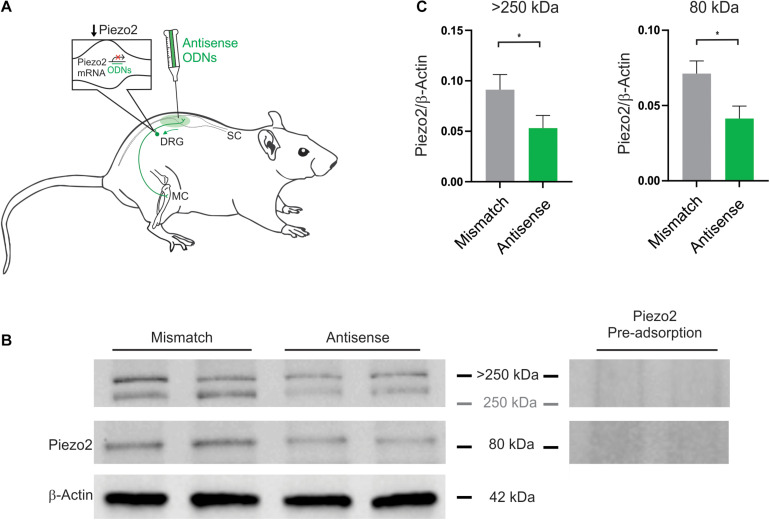
Piezo2 knockdown approach and confirmation of protein knockdown. **(A)** Piezo2 antisense or mismatch oligodeoxynucleotides (ODNs) were administered by intrathecal injection. **(B)** Western blot analysis revealed that the intensity of the bands >250 kDa, and at 80 kDa, are significantly reduced by administration of Piezo2 antisense, relative to mismatch ODNs. Pre-adsorption of the Piezo2 antibody with the manufacturer’s peptide (Piezo2 antibody blocking peptide, Novus Biologicals, #NBP1-78624PEP) at 5 μg/ml completely abolishes Piezo2 protein bands at >250 kDa, and at 80 kDa, in rat DRG. **(C)** Densitometry analysis revealed a significant reduction in the ratio of Piezo2/β-Actin in the DRG of animals injected with Piezo2 antisense ODNs (*n* = 6) relative to mismatch control ODNs (*n* = 6). Data represents mean ± SEM, *P < 0.05, unpaired *t*-test.

Selective antagonists or function blocking antibodies that directly target Piezo2 are not yet available, and we are currently unable to make recordings from bone afferent neurons in mice so cannot take advantage of transgenic approaches to manipulation of Piezo2. Instead, we used Piezo2 knockdown with antisense oligodeoxynucleotides (ODNs) to explore roles for Piezo2 in regulating the mechanical sensitivity of bone afferent neurons in rats (Figure 1A). The approach of using antisense ODNs to attenuate the expression of proteins in peripheral nociceptors is well supported in the literature (Khasar et al., 1996; Lai et al., 2002; Summer et al., 2006; Chien et al., 2007; Bogen et al., 2008, 2012; Ferrari et al., 2020). It has been previously used to knock down Piezo2 expression in the lumbar DRG of rats (Ferrari et al., 2015), and also results in a significant reduction in Piezo2 mRNA in DRG (Eijkelkamp et al., 2013) and chondrocytes (Du et al., 2020) taken from mice. In order to maximize the efficacy of this method, we used a mixture of 3 different ODNs to rat Piezo2 mRNA (Ferrari et al., 2015): 5′-CCACCACATAAACACCTGC-3′, 5′-TTCCTCCTCTTCACTATCCG-3′ and 5′-CCTCAATGG TTTCCGTAGTTC-3′ (Sigma-Aldrich, St Louis, Missouri, United States). For control experiments, a mixture of 3 similar ODNs were used, that contained specific bases that were a mismatch to the reported rat Piezo2 sequence: 5′-ACATCAC ACGAACTCCAGC-3′, 5′-GT CATCGTCATCACATTGCG-3′ and 5′-TCTCAGTGCTCTCCATAGGTA-3′ (Sigma-Aldrich, St Louis, Missouri, United States, mismatched bases underlined). The antisense ODNs showed 100% identity with only the rat mRNA sequence for Piezo2, but no other known genes, whereas the mismatch ODNs did not fully align with any known rat gene^1^. The mixtures of antisense or mismatch ODNs were administered intrathecally, in a volume of 20 μl, once daily for three consecutive days, at a dose of 0.35 or 3.5 μg/μl. We did not observe any differences in the function of bone afferent neurons between the two doses, so we report data from the two doses together as a single group.

Animals were anesthetized with isoflurane (4% induction; 2.5% maintenance). Intrathecal injections of the ODNs were made into the lumbar subarachnoid space with a Hamilton syringe, between L4 and L5 vertebra, once per day for three days before we performed electrophysiological experiments. Correct placement of the intrathecal injection was confirmed by the presence of a sudden tail flick or rapid limb flinch, both reflexes that occur in response to mechanical stimulation to the lumbar subarachnoid space. Recovery was monitored continuously until the animal was conscious and freely moving without any noticeable disturbances (usually after a few minutes).

### Electrophysiological Recordings Using an *in vivo* Bone–Nerve Preparation

The day after the third intrathecal injection of Piezo2 ODNs, rats were anesthetized with urethane (50% w/v, 1.5 g/kg; i.p.), and electrophysiological experiments were made using our recently developed *in vivo*, bone nerve preparation (Nencini and Ivanusic, 2017; Nencini et al., 2017, 2018, 2019; Morgan et al., 2019). A fine branch of the tibial nerve that innervates the marrow cavity of the rat tibia was dissected and placed over a platinum hook electrode for electrophysiological recordings. Whole-nerve electrical activity was amplified (1000X) and filtered (high-pass 100 Hz, low pass 3 kHz) using a differential amplifier (DP-311, Warner Instruments), sampled at 20 kHz (PowerLab, AD Instruments, Australia) and stored to PC using the recording software LabChart (AD Instruments; RRID: SCR_017551). Mechanical stimulation of the axon terminals of bone afferent neurons innervating the marrow cavity was achieved by injecting heparinized physiological saline (0.9% sodium chloride) into the marrow cavity through a needle that was connected to a feedback-controlled syringe pump (PHD ULTRA Pump, Harvard Apparatus) via polyethylene tubing. Changes in intraosseous pressure were measured using a bridge-amplified (TAM-D Amplifier, Harvard Apparatus) signal derived from a pressure transducer (APT300 Transducer, Harvard Apparatus) placed to measure the input pressure to the bone. The pump uses this as feedback to adjust flow through the system to control and maintain constant input pressures. We used this feature to apply a ramp-and-hold mechanical stimulus with an initial flow rate of 7 ml/min during the ramp phase (3 s), and a constant 300 mmHg of pressure delivered during the hold phase (15 s). These data were stored to PC in parallel with the nerve recordings.

All spikes with positive and/or negative peaks clearly above noise were sampled from whole-nerve recordings. We were unable to routinely record conduction velocities in each experiment because we could not electrically stimulate the receptive fields of individual units buried deep inside the marrow cavity. Instead, we classified spikes as originating from C, Aδ or Aβ units on the basis of previously published experiments, using the same recording configuration, in which we demonstrated a linear relationship between conduction velocity and peak-to-peak action potential amplitude for units activated with mechanical stimulation from within the bone marrow (Nencini and Ivanusic, 2017; Nencini et al., 2018). In this previous work, impulses with amplitudes <40 μV were defined as originating from C fibers (conduction velocities <2.5 m/s) and those with amplitudes between 40 and 145 μV were defined as originating from Aδ fibers (conduction velocities between 2.5 and 12.5 m/sec). For a thorough discussion of how this division was selected, and to view the data that supports this classification, see Nencini et al. (2018). Mechanically evoked action potentials originating from single Aδ bone afferent neurons were discriminated from whole-nerve recordings according to their amplitude and duration using Spike Histogram software (LabChart 8, AD Instruments; RRID:SCR_017551). We excluded units from analysis if their amplitude and duration changed over the course of the recording. We were not able to isolate individual C bone afferent neurons using our recording configuration so do not report on C fiber bone afferent neurons in this study. In all cases, we use small “n” to represent the number of units and capital “N” to represent the number of hind-limb preparations recorded from.

To test whether Piezo2 knockdown alters the response of Aδ bone afferent neurons to mechanical stimulation, we compared their responses to the mechanical stimulus (discharge frequency and threshold for activation), in animals injected with either Piezo2 antisense or mismatch ODNs. Discharge frequency was reported over the entire ramp-and-hold pressure stimulus (total discharge frequency), for the ramp phase of the stimulus (defined as the first 3 s of the ramp-and-hold pressure stimulus), and for the hold phase of the stimulus (defined as the 5 s period starting 5 s after the beginning of the hold phase of the pressure stimulus). Threshold for activation was assessed on the rising phase of the pressure ramp. We also explored the responses of Aδ bone afferent neurons to pairs of 300 mmHg ramp-and-hold pressure stimuli delivered using two different inter-stimulus time intervals (ISIs): 15 and 30 min. To assess the conditioning effect of the ISIs, the threshold for activation and total discharge frequency at the second stimulus was presented as a percentage of that at the first (baseline) stimulus.

To investigate whether Piezo2 contributes to mechanical sensitization of bone afferent neurons, we tested whether NGF-induced sensitization of Aδ bone afferent neurons was affected by Piezo2 knockdown. We have previously reported that NGF injected directly into the marrow cavity in our bone-nerve recordings induces a rapid and transient increase in the mechanical sensitivity of Aδ bone afferent neurons, that peaks at 15 min after injection, and resolves within 30 min (Nencini et al., 2017). In this experiment, we made recordings from animals (either Piezo2 knockdown or mismatch control) that received an injection of NGF (5 μg in 10 μl; Sigma-Aldrich, #N0513) directly into the marrow cavity through a needle using a Hamilton syringe attached with polyethylene tubing. The concentration of NGF used is in the range that produces behavioral pain-like responses when applied to the footpad (Mills et al., 2013) and the tibial marrow cavity (Nencini et al., 2017) of naïve rats. Responses of the Aδ bone afferent neurons to the ramp-and-hold pressure stimulus were assessed 15 min before, and 15 min after application of NGF. This 30 min ISI was used to avoid the effect of stimulus-evoked fatigue on discharge frequency observed with 15 min ISIs in Piezo2 knockdown animals. The thresholds for mechanical activation and the discharge frequency during the pressure stimulus were determined following injection of NGF and expressed as a percentage of pre-NGF injection (baseline) values. Decreases of more than 20% in the threshold for mechanical activation, relative to pre-NGF injection values, were used as indicators of sensitization (Nencini et al., 2017). The number of NGF sensitized vs. non-sensitized units were compared between animals injected with Piezo2 antisense and mismatch ODNs.

To exclude the possibility that the mismatch ODNs themselves might have unpredicted effects on the function of Aδ bone afferent neurons, we also compared responses between animals injected with mismatch ODNs or naïve control animals. The naïve control animal data for the response of Aδ bone afferent neurons to mechanical stimulation were acquired from experiments previously reported in Figure 8 of Nencini and Ivanusic (2017). The naïve control animal data for NGF-induced sensitization of Aδ bone afferent neurons were acquired from experiments previously reported in Figure 4 of Nencini et al. (2017). These data were re-analyzed for the purpose of the present study using the approaches outlined above.

Statistical analyses of electrophysiological data were performed with Prism (GraphPad Software; RRID:SCR_002798). To avoid errors related to pseudo replication of electrophysiological data that included multiple cells derived from a single recording, statistical significance between treatment groups was evaluated using a mixed model nested one-way ANOVA (naïve v mismatch v antisense), followed by Dunnett’s *post hoc* analysis only if the mixed model reported significant effects. An ANOVA was used to test for differences in the number of units recorded in each experiment (naïve v mismatch v antisense). The chi-square test (with Bonferroni’s adjustment for multiple comparisons when required) was used to test for differences in the proportion of sensitized vs. non-sensitized bone afferent neurons in the different treatment groups. In all cases, *P* < 0.05 was used to define statistical significance.

### Western Blot

We have used a validated Piezo2 antibody (Novus Biologicals, #NBP1-78624; RRID: AB_11005294; see antibody specificity below), in Western blot analysis, to confirm that Piezo2 antisense ODN treatment reduced Piezo2 protein, relative to mismatch ODN treatment, in the lumbar DRG of rats. For this purpose, rats that were injected with either Piezo2 mismatch (*n* = 6) or antisense (*n* = 6) ODNs, were anesthetized with ketamine/xylazine (ketamine 130 mg/kg, xylazine 10 mg/kg; i.p.) and killed by exsanguination, 6 h after the third intrathecal injection of ODNs. Lumbar DRG L3-L5 were quickly removed and sonicated in ice-cold RIPA buffer (50 mM Tris-HCl, pH 7.4, 150 mM NaCl, 1% Triton X-100, 0.1% SDS, 1 mM EDTA) containing protease inhibitor cocktail (Roche Diagnostics). Lysates were constantly agitated at 4°C for 1 h and then centrifuged at 12,000 rpm for 10 min (4°C) and the supernatants collected. The total protein concentration was measured using the Pierce BCA protein assay kit (Life Technologies). Equal amounts of protein (50 μg) were boiled at 95°C for 5 min in Laemmli sample buffer (65.8 mM Tris-HCl, pH 6.8, 26.3% glycerol, 2.1% SDS, 0.01% bromophenol blue) containing 2-mercaptoethanol. The protein lysates were then separated by SDS-PAGE and transferred onto a 0.45 μm PVDF membrane (Bio-Rad). The membrane was blocked for 1 h at room temperature in TBST (20mM Tris-HCl, pH 8, 150mM NaCl, 0.05% Tween-20) containing 5% non-fat skim-milk, and then incubated with rabbit anti-Piezo2 antibody (1:1000; Novus Biologicals, #NBP1-78624; RRID: AB_11005294) overnight at 4°C. The membrane was washed 3 times with TBST and incubated with goat anti-rabbit HRP-conjugated secondary antibody (1:5000; Cell Signaling Technology, #7074S) for 1 h at room temperature. Following another 3 washes in TBST, immunoreactive protein bands were visualized using enhanced chemiluminescence (Clarity ECL, Bio-Rad). The membrane was re-probed with a mouse anti-β-actin antibody (1:1000; Sigma-Aldrich, #A5441; RRID: AB_476744), and incubated with a horse anti-mouse HRP-conjugated secondary antibody (1:5000; Cell Signaling Technology, #7076S). All antisera were diluted in TBST containing 5% BSA. Molecular weight was estimated using the Precision Plus Protein All Blue Standards (Bio-Rad). Results were analyzed using computer-assisted densitometry (ImageLab Software v6.1, Bio-Rad; RRID: SCR_014210). The density of each band was expressed as arbitrary units. The Piezo2 band density was normalized to β-actin, for each animal, and compared between Piezo2 antisense and mismatch treated animals using an unpaired *t*-test in Prism (GraphPad Software; RRID: SCR_002798).

### Retrograde Tracing and Immunohistochemistry

Animals were anesthetized with isoflurane (4% induction; 2.5% maintenance). A skin incision was made on the medial aspect of the tibia and a small hole was made in the cortical bone on the medial aspect of the tibial diaphysis using a sterile needle. A Hamilton syringe was used to inject the retrograde tracer Fast Blue (2 μl FB; 10% in dH_2_O) through the hole and directly into the marrow cavity. The hole was sealed with bone wax to prevent leakage into surrounding tissues. The area was washed extensively with 0.1M phosphate buffered saline (pH 7.4; PBS) and inspected to ensure there was no leakage to surrounding tissues. Skin incisions were closed. Animals were left for a 10-day survival period to allow for transport of the tracer to neuronal cell bodies in the DRG. Each animal was given an overdose of ketamine/xylazine (ketamine 130 mg/kg, xylazine 10 mg/kg; i.p.) and was perfused via the ascending aorta with 500 ml of heparinized PBS followed by 500 ml of 4% paraformaldehyde in PBS. Lumbar DRG L3 were dissected and left overnight in 30% PBS-sucrose, frozen in liquid nitrogen cooled isopentane, and were sectioned at 20 μm using a cryostat the next day. Multiple series of sections were collected on gelatinized glass slides (0.1% chrome alum and 0.5% gelatin) and processed for immuno-labeling. Sections were immuno-labeled to determine whether retrograde labeled bone afferent neurons expressed Piezo2 (1:100; polyclonal rabbit anti-Piezo2; Novus Biologicals, #NBP1-78624; RRID: AB_11005294) and/or TrkA(high affinity receptor for NGF; 1:500; polyclonal goat anti-TrkA; R&D Systems, #AF1056;RRID: _AB_2283049). The concentration of each of the primary antibodies was optimized in preliminary experiments. All antisera were diluted in PBS containing 0.3% Triton X-100 and 0.1% sodium azide. Sections were washed 3 times in PBS, blocked in 10% normal horse serum for 1 h, and incubated in the primary antisera at room temperature overnight. Following three further washes in PBS, they were incubated in secondary antibody for 2 h, washed 3 times in PBS, and cover-slipped using DAKO fluorescence mounting medium. Details of the primary and secondary antibodies used are provided in Table 1.

**TABLE 1:** Details of the primary and secondary antibodies used for immunohistochemistry.

	Immunogen	Manufacturer	Dilution
**Primary antibody**			
Rabbit α Piezo2	Human Piezo2 protein (residues 1600-1650)	Novus Biologicals, CO, United States; Rabbit polyclonal; #NBP1-78624	1:100
Goat α TrkA	Mouse myeloma cell line NS0-derived recombinant rat TrkA (Ala33-Pro418)	R&D Systems, MN, United States; Goat polyclonal; #AF1056	1:500
**Secondary antibody**			
Donkey α Rabbit Alexa594		Molecular Probes, Invitrogen; #A21207	1:200
Donkey α Goat Alexa488		Molecular Probes, Invitrogen; #A11055	1:200

Sections of DRG were examined and photographed with a 10x objective using a Zeiss Axioskop fluorescence microscope (Carl Zeiss, Oberkochen, Germany) fitted with an AxioCamMRm camera. FITC, Texas Red and UV filter sets were used to discriminate labeling with the Alexa Fluor 488 and 594 fluorophores, and Fast Blue, respectively. Counts and soma size measurements (cross-sectional area of soma) were made directly from the images using Zen lite software (Zen 2011, Carl Zeiss; RRID: SCR_013672; Oberkochen, Germany). To avoid double counting, and to sample from the widest part of the cell, only cells with a nucleus visible under the microscope were examined. The counts presented are estimates of the total number of bone afferent neurons and may overestimate afferents with large cell bodies. We determined the proportion of retrograde labeled neurons that expressed each antibody marker for each animal. Figures were prepared using CorelDraw software (CorelDraw Graphics Suite; RRID: SCR_014235; Ottawa, Canada). Individual images were contrast and brightness adjusted. No other manipulations were made to the images.

### Piezo2 Antibody Specificity

The Piezo2 antibody (polyclonal rabbit anti-Piezo2, Novus Biologicals, #NBP1-78624; RRID: AB_11005294) used in Western blot analyses and immuno-labeling was raised against a synthetic peptide made to an internal portion of the human PIEZO2 protein (between residues 1600–1650) [UniProt# Q9H5I5]. UniProt describes 4 isoforms of human PIEZO2 produced by alternative splicing: 318.1, 311.7, 80.8, 320.9 kDa. The ability of this antibody to detect Piezo2 has been confirmed by antibody-mediated affinity purification of native Piezo2 from mouse DRG, followed by mass spectrometry and label-free quantification (Narayanan et al., 2016). Piezo2 was detected in samples treated with the Piezo2 antibody, but not in samples treated with its isotype control antibody (Narayanan et al., 2016). In addition, Piezo2 immuno-staining of mesencephalic trigeminal neurons with this antibody was abolished by AAV-shRNA mediated knockdown of Piezo2 in C57B1/6J mice, and in Pvalb-Cre:RCE:Piezo2cKO mice, but not in mice delivered scrambled shRNA or in wild type littermate control mice (Florez-Paz et al., 2016). Western blot analysis reveals the antibody labels an 80 kDa band in mice, and that the intensity of the band is significantly reduced by siRNA treatment targeted specifically at Piezo2 (Du et al., 2020). The 80 kDa band has also been reported (using a different antibody) in human vascular endothelial cells and rat bladder tissue, and siRNA mediated knockdown of this band is associated with reduced calcium signaling in human vascular endothelial cells (Yang et al., 2016a). In the current study, we have further shown that pre-adsorption of the antibody with the manufacturer’s peptide (Piezo2 antibody blocking peptide, Novus Biologicals, #NBP1-78624PEP) at 5 μg/ml completely abolishes bands at >250 kDa and 80 kDa in rat DRG (Figure 1B and Supplementary Figure), and that the intensity of the same bands are significantly reduced by antisense treatment targeted specifically at Piezo2 (Figure 1C).

## Results

### Confirmation of Piezo2 Knockdown

Piezo2 knockdown in the DRG was achieved with intrathecal delivery of antisense oligodeoxynucleotides (ODNs) to the dorsal roots of lumbar DRG (Figure 1A). Knockdown of Piezo2 expression was demonstrated by Western blot, using DRG taken from ODN-treated rats (Figure 1B). Western blot analysis revealed a reduction in the density of Piezo2 protein bands at >250 kDa, and at 80 kDa, in DRG lysates taken from animals treated with Piezo2 antisense ODNs, relative to those treated with mismatch ODNs (Figure 1C; unpaired *t*-test, *n* = 6 animals/group, *P* < 0.05).

### Piezo2 Knockdown Alters the Response of Aδ Bone Afferent Neurons to Noxious Intraosseous Pressure

**FIGURE 2:**
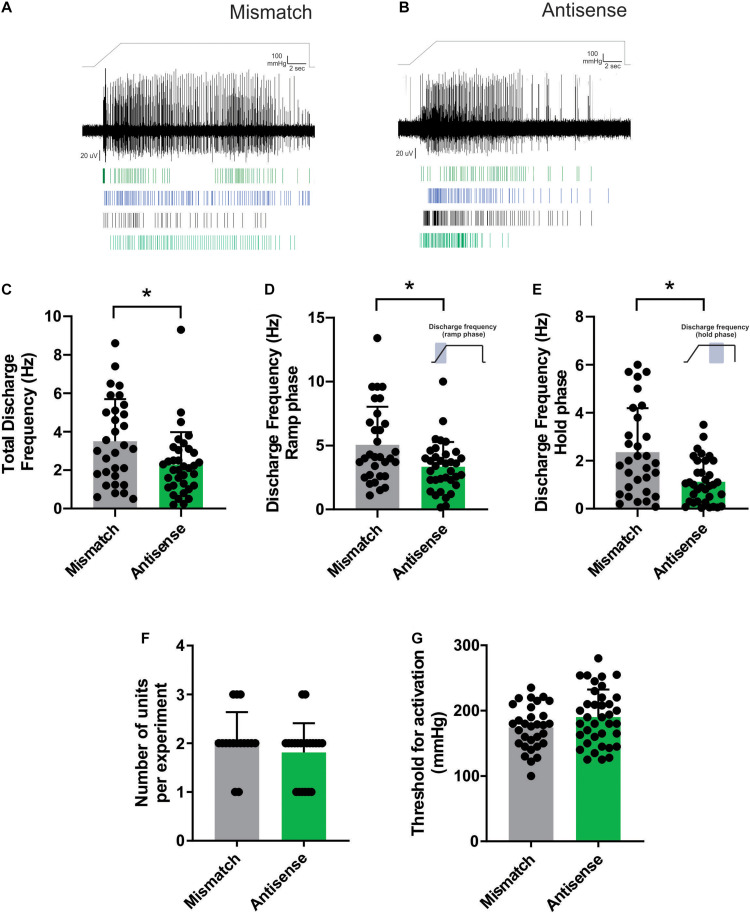
Piezo2 knockdown alters the response of Aδ bone afferent neurons to noxious mechanical stimulation. **(A,B)** Examples of whole-nerve recordings, and rasters of single unit activity, from Piezo2 mismatch **(A)** and antisense **(B)** ODN treated animals. **(C)** There was a significantly reduced total discharge frequency in Aδ bone afferent neurons recorded from Piezo2 antisense treated animals compared to mismatch control animals [mixed model, *F* (3.278), DFn (2), Dfd (43), *Dunnett’s *P* < 0.05; *n* = 23 naïve/31 mismatch/35 antisense, *N* = 10 naïve/15 mismatch/21 antisense]. The reduction in discharge frequency was observed during both the **(D)** ramp [F (3.641), DFn (2), Dfd (43), Dunnett’s *P* < 0.05; *n* = 23 naïve/31 mismatch/35 antisense, *N* = 10 naïve/15 mismatch/21 antisense] and the **(E)** hold phase of the pressure stimulus [F (5.757), DFn (2), Dfd (43), Dunnett’s *P* < 0.05; *n* = 23 naïve/31 mismatch/35 antisense, *N* = 10 naïve/15 mismatch/21 antisense]. **(F)** There was no difference in the number of Aδ bone afferent neurons isolated per experiment in recordings made from Piezo2 knockdown compared to mismatch control animals (*P* > 0.05, unpaired *t*-test, *n* = 33 mismatch/38 antisense, *N* = 16 mismatch/21 antisense). **(G)** There was no difference in the threshold for mechanical activation of Aδ bone afferent neurons in recordings made from Piezo2 knockdown compared to mismatch control animals [mixed model, F (6.139), DFn (2), Dfd (45), Dunnett’s *P* > 0.05; *n* = 23 naïve/31 mismatch/35 antisense, *N* = 10 naïve/15 mismatch/21 antisense].

Aδ bone afferent neurons recorded from animals administered Piezo2 antisense ODNs had reduced activity in response to pressure applied to the marrow cavity, compared to those recorded from mismatch control animals (Figures 2A,B). Mixed model analysis revealed significantly reduced discharge frequency calculated over the entire duration of the ramp-and-hold pressure stimulus (total discharge frequency), in animals administered Piezo2 antisense ODNs relative to those administered mismatch ODNs [*F* (3.278), DFn (2), Dfd (43), Dunnett’s *P* < 0.05; *n* = 23 naïve/31 mismatch/35 antisense, *N* = 10 naïve/15 mismatch/21 antisense; Figure 2C]. To determine if the reduced activity was confined to either the ramp phase when the pressure was changing, or the hold phase of the stimulus when pressure was constant, we repeated the analyses using data that were confined to each of these distinct parts of the ramp-and-hold pressure stimulus we applied. Mixed model analyses revealed a significant reduction in discharge frequency during both the ramp [F (3.641), DFn (2), Dfd (43), Dunnett’s *P* < 0.05; *n* = 23 naïve/31 mismatch/35 antisense, *N* = 10 naïve/15 mismatch/21 antisense; Figure 2D] and the hold phase of the pressure stimulus [F (5.757), DFn (2), Dfd (43), Dunnett’s *P* < 0.05; *n* = 23 naïve/31 mismatch/35 antisense, *N* = 10 naïve/15 mismatch/21 antisense; Figure 2E]. There were no differences in the number of discriminable Aδ bone afferent units responding to the pressure stimulus [ANOVA, *P* > 0.05, *n* = 23naïve/31 mismatch/*n* = 35 antisense, *N* = 10 naïve/15 mismatch/21 antisense; Figure 2F], or their threshold for mechanical activation [mixed model, F (6.139), DFn(2), Dfd(45), Dunnett’s *P* > 0.05; *n* = 23 naïve/31 mismatch/35 antisense, N = 10 naïve/15 mismatch/21 antisense; Figure 2G], in recordings made from Piezo2 knockdown compared to mismatch control animals. Importantly, the same mixed model analyses revealed no differences in discharge frequency or threshold for activation between naïve animals and those administered mismatch ODNs (data not shown). Whilst the data clearly show that manipulating Piezo2 protein levels can affect the activity of mechanically sensitive Aδ bone afferent neurons, it is important to acknowledge that our approach did not knockout Piezo2 entirely (Figure 1). It is possible that a more complete knockdown of Piezo2 is required for effects to be observed on the threshold for activation of Aδ bone afferent neurons.

### Piezo2 Knockdown Alters the Response of Aδ Bone Afferent Neurons to Repetitive Mechanical Stimulation

**FIGURE 3:**
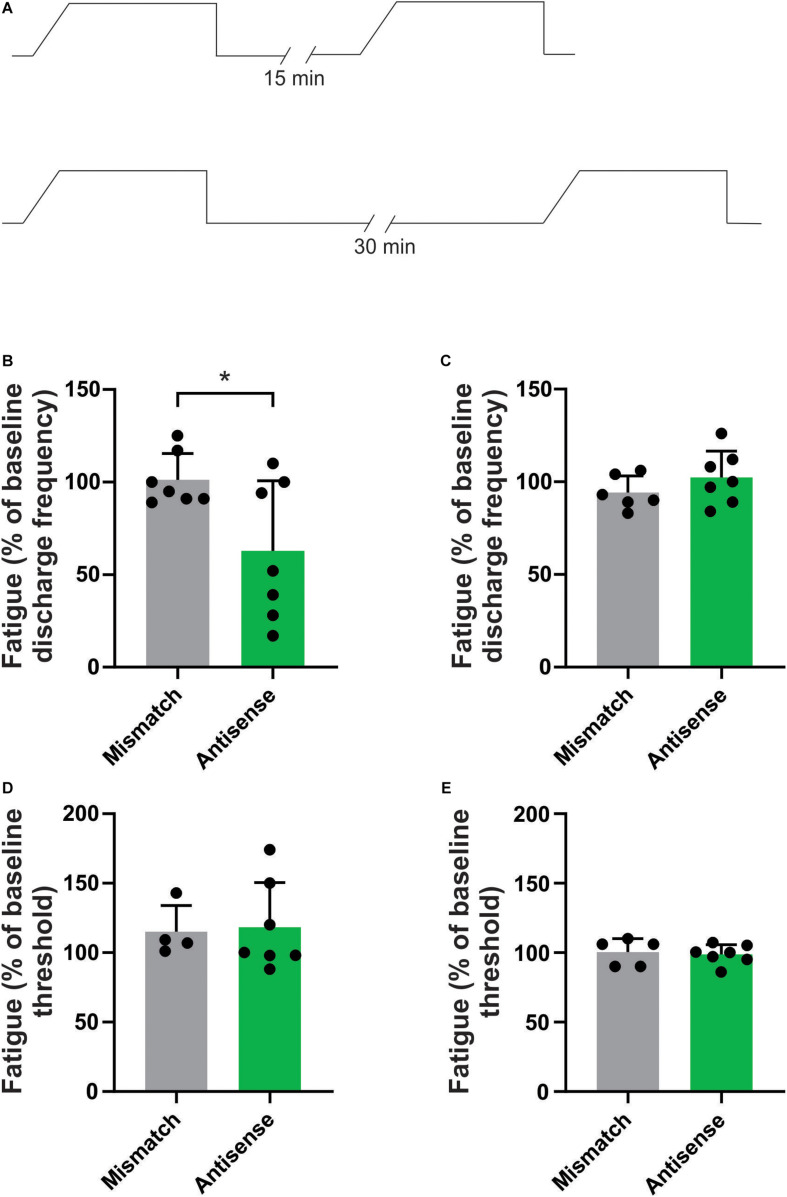
Piezo2 knockdown prolongs stimulus evoked fatigue. **(A)** Schematic representation of the repetitive stimulation experimental protocol. The response to repetitive stimulation was measured at 15 and 30 min interstimulus intervals (ISIs) and compared between Piezo2 antisense and mismatch control treated animals. **(B,C)** There was a significant decrease in the discharge frequency of Aδ bone afferent neurons recorded from animals treated with Piezo2 antisense, relative to those treated with mismatch control, at 15 min (**B**, *P < 0.05, unpaired *t*-test, *n* = 7 mismatch/7 antisense, *N* = 3 mismatch/4 antisense) but not 30 min (**C**, *P* > 0.05, unpaired *t*-test, *n* = 6 mismatch/7 antisense, *N* = 3 mismatch/4 antisense) ISIs. **(D,E)** There were no differences in the threshold for activation of Aδ bone afferent neurons recorded from animals treated with Piezo2 antisense, relative to those treated with mismatch control, at either 15 min (**D**, *P* > 0.05, unpaired *t*-test, *n* = 4 mismatch/6 antisense, *N* = 3 mismatch/4 antisense) or 30 min (**E**, *P* > 0.05, unpaired *t*-test, *n* = 5 mismatch/7 antisense, *N* = 3 mismatch/4 antisense) ISIs.

We have previously reported that repetitive mechanical stimulation can lead to significant changes in the response properties of bone afferent neurons (Nencini and Ivanusic, 2017). Specifically, we documented stimulus-evoked fatigue of Aδ bone afferent neurons in response to prior stimulation if the ISI was less than 10 min. To test whether Piezo2 knockdown altered the response of Aδ bone afferent neurons to repetitive mechanical stimulation, we assayed their discharge frequency and threshold for activation in response to repeated application of the ramp-and-hold pressure stimulus, at ISIs of either 15 or 30 min (Figure 3A). There was a significant reduction in the discharge frequency of Aδ bone afferent neurons recorded from Piezo2 knockdown animals, relative to those recorded from mismatch control animals, in response to mechanical stimuli delivered at intervals of 15 (unpaired *t*-test, *P* < 0.05, *n* = 7 mismatch/7 antisense, *N* = 3 mismatch/4 antisense; Figure 3B) but not 30 min (unpaired *t*-test, *P* > 0.05, *n* = 6 mismatch/7 antisense, *N* = 3 mismatch/4 antisense; Figure 3C). There was no change in the threshold for activation of Aδ bone afferent neurons recorded from Piezo2 knockdown animals, relative to those recorded from mismatch control, at either 15 (unpaired *t*-tests, *P* > 0.05, *n* = 4 mismatch/6 antisense, *N* = 3 mismatch/4 antisense; Figure 3D) or 30 min (unpaired *t*-tests, *P* > 0.05, *n* = 5 mismatch/7 antisense, *N* = 3 mismatch/4 antisense; Figure 3E) ISIs. Thus most units recorded from animals administered Piezo2 antisense ODNs took longer to recover from stimulus-evoked fatigue than those recorded from mismatch control animals, or in naïve animals in our previous study.

### Piezo2 Knockdown Prevents NGF-Induced Sensitization of Aδ Bone Afferent Neurons to Mechanical Stimulation

**FIGURE 4:**
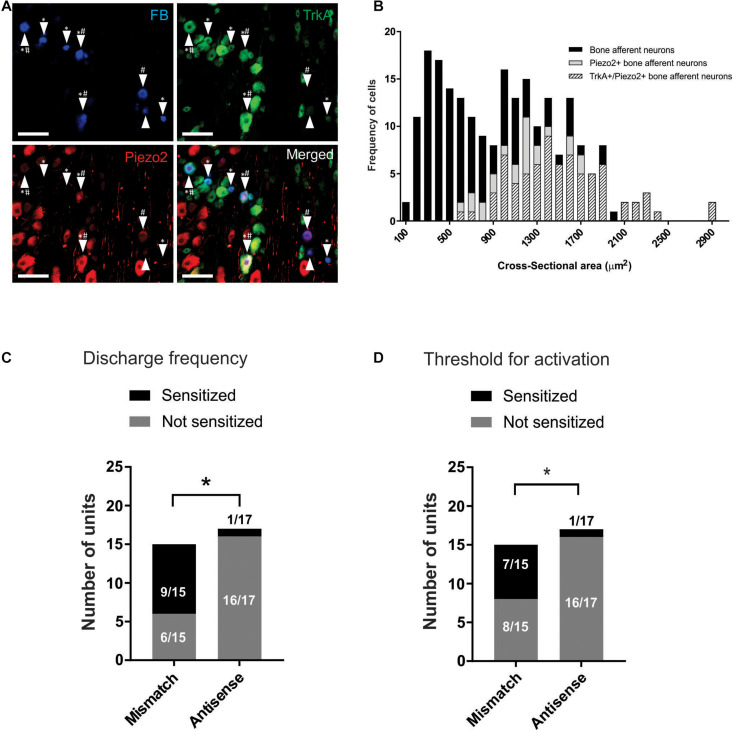
Piezo2 knockdown prevents NGF-induced sensitization of Aδ bone afferent neurons. **(A)** Retrograde labeling of bone afferent neurons (FB, blue, arrowheads) revealed that a substantial proportion of neurons that innervate bone express Piezo2 (red, hashes) and the NGF receptor TrkA (green, asterisks). Scale bars = 100 μm. **(B)** Frequency histogram showing that the majority of medium-sized bone afferent neurons expressed Piezo2, and that most Piezo2 expressing bone afferent neurons also expressed TrkA. **(C)** A significantly lower proportion of Aδ bone afferent neurons were sensitized (increased discharge frequencies) by NGF in Piezo2 antisense treated animals compared to mismatch control animals [chi-square test, *X*^2^ (1, *N* = 32) = 10.86,*P < 0.05, *n* = 15 mismatch/17 antisense, *N* = 8 mismatch/9 antisense]. **(D)** A significantly lower proportion of Aδ bone afferent neurons were sensitized (lower thresholds for activation) by NGF in Piezo2 antisense treated animals compared to mismatch control animals (chi-square test, *X*^2^ (1, *N* = 32) = 7.069,*P < 0.05, *n* = 15 mismatch/17 antisense, *N* = 8 mismatch/9 antisense).

We used retrograde tracing and immunohistochemistry to determine if Piezo2 expressing bone afferent neurons co-express the NGF receptor TrkA (Figure 4A). A total of 222 retrograde labeled bone afferent neurons were counted in L3 DRG taken from three animals. A substantial proportion of bone afferent neurons expressed Piezo2 (44 ± 3.3%; *n* = 3 animals), and most of these also expressed TrkA (77 ± 7.8%; *n* = 3 animals) (Figure 4B). These findings show that a substantial proportion of bone afferent neurons express both Piezo2 and the NGF receptor TrkA, and provide the necessary substrate for an interaction between Piezo2 and NGF to contribute to pain signaling.

To investigate whether Piezo2 contributes to sensitization of Aδ bone afferent neurons, and if there is a functional interaction between Piezo2 and NGF in Aδ bone afferent neurons, we then tested whether NGF-induced sensitization of Aδ bone afferent neurons to mechanical stimulation was affected by Piezo2 knockdown. We classified units as sensitized by NGF if their discharge frequency was increased by more than 20%, and/or their threshold for activation was reduced by more than 20%, relative to the discharge frequency or threshold for activation of the same unit before NGF was injected. There was a significant reduction in the proportion of units that were sensitized, relative to those that were not sensitized, in animals administered Piezo2 antisense compared to mismatch ODNs (chi-square test, discharge frequency: *X*^2^ (1, *N* = 32) = 10.86, *P* < 0.05, Figure 4C; threshold for activation: *X*^2^ (1, *N* = 32) = 7.069, *P* < 0.05, Figure 4D; *n* = 15 mismatch/17 antisense, *N* = 8 mismatch/9 antisense). Piezo2 knockdown almost entirely prevented NGF-induced sensitization defined by these criteria (only 1/17 units was sensitized by NGF in recordings made from animals administered antisense ODNs) (Figures 4C,D). The proportion of units that were sensitized, compared to those that were not, did not differ between animals administered mismatch ODNs and naïve animals [chi-square test, discharge frequency: *X*^2^ (1, *N* = 32) = 0.1271 *P* > 0.05; threshold for activation: *X*^2^ (1, *N* = 32) = 0.6193, *P* > 0.05; *n* = 15 mismatch/17 antisense, *N* = 8 mismatch/9 antisense].

## Discussion

The main findings of the present study are that Piezo2 contributes to the response of Aδ bone afferent neurons to noxious mechanical stimulation, and that interactions with NGF are likely to be important for its function.

Destruction of bone by osteolytic processes or trauma can lead to mechanical injury or distortion of bone that activates mechanically sensitive nerve terminals in bone marrow to produce pain (Haegerstam, 2001; Bove et al., 2009; Mantyh, 2014). Bone cancers, fractures, intra-osseous engorgement syndrome, osteoarthritis and osteomyelitis can further produce inflammation and/or an increase in intra-osseous pressure that compresses mechanically sensitive nerve terminals in bone marrow to produce pain (Lemperg and Arnoldi, 1978; Arnoldi et al., 1980; Haegerstam, 2001; Kidd et al., 2004; Urch, 2004; Starr et al., 2008; Mantyh, 2014). Agents that are known to inhibit inflammatory processes reduce mechanically induced pain in animal models of bone cancer (Honore and Mantyh, 2000) and pro-inflammatory cytokines contribute to mechanically induced pain in bone fracture models (Li et al., 2009). These studies highlight mechanical disturbances of the bone marrow as important components of bone pathology, and suggest that treatment strategies targeted specifically at mechanically induced pain will provide therapeutic benefit.

Bone pain is transmitted by two main classes of peripheral nociceptors (Nencini and Ivanusic, 2016). Aδ nociceptors are myelinated sensory neurons that transmit fast, intense pain that is relevant to fracture, acute inflammation or mechanical instability of bone. C nociceptors are unmyelinated sensory neurons that encode slow, aching pain relevant to more chronic conditions such as osteoarthritis or bone cancer. Both Aδ and C fiber sensory neurons innervate the bone marrow (Furusawa, 1970; Seike, 1976; Mach et al., 2002; Ivanusic et al., 2006; Ivanusic, 2009; Ishida et al., 2016) and are responsive to noxious chemical and mechanical stimuli (Furusawa, 1970; Sakada and Taguchi, 1971; Seike, 1976; Ivanusic et al., 2005; Nencini and Ivanusic, 2017; Nencini et al., 2017). Aδ bone afferent neurons have been classified as one of two types on the basis of the way they adapt to noxious mechanical stimulation applied to the bone marrow (Nencini and Ivanusic, 2017). Phasic-tonic units respond best to the intensity of sustained intra-osseous pressure and may signal pain associated with pathologies that involve sustained increases in pressure within bone, for example intra-osseous engorgement syndrome. In contrast, phasic units respond best to the rate of change in intra-osseous pressure and are likely to signal pain associated with rapid changes in pressure within the marrow cavity, for example during needle aspiration of bone marrow or emergency intra-osseous vascular access. The response of single bone afferent neurons with C fiber conduction velocities has not yet been reported, but there is clear evidence that C bone afferent neurons in whole-nerve recordings can be activated by noxious mechanical stimuli applied to bone marrow (Nencini et al., 2018, 2019; Morgan et al., 2019). The identity of the channels that impart mechanical sensitivity to Aδ and C bone afferent neurons, and therefore contribute to mechanically induced bone pain, has not been studied before.

In the present study, we have shown that Piezo2 may not be required for Aδ bone afferent neurons to respond to noxious mechanical stimulation, but that it is necessary to maintain their activity during noxious mechanical stimulation of the marrow cavity. This is consistent with reports that Piezo2 knockout does not prevent nociceptors from transducing mechanical stimuli, but that it does reduce the sensitivity of Aδ mechano-nociceptors in the skin-nerve preparation (Ranade et al., 2014; Murthy et al., 2018). Interestingly, one of these two studies also reports effects of Piezo2 knockout on C nociceptors in the skin-nerve preparation (Murthy et al., 2018), while the other does not (Ranade et al., 2014). This discrepancy may be related to the efficiency of Piezo2 ablation in the two different Piezo2 knockout mouse lines used. Given that Aδ bone afferent neurons mediate fast, intense pain, of the sort experienced in response to fracture, acute inflammation or mechanical instability of bone, our findings suggest that manipulation of Piezo2 signaling might be useful to reduce pain associated with these conditions. The altered responses of Aδ bone afferent neurons to repetitive stimulation further suggests that Piezo2 contributes to stimulus evoked fatigue in these neurons. Our findings provide the first evidence that Piezo2 contributes to pain signaling in sensory neurons that innervate the bone.

Sensitization of peripheral bone nociceptors has been used to explain increased sensitivity of patients to mechanical stimuli in a variety of bony pathologies (Portenoy et al., 1999; Honore and Mantyh, 2000; Haegerstam, 2001). Sensitized peripheral nociceptors have reduced thresholds for activation and increased activity in response to a given stimulus. This renders them more sensitive to noxious stimuli, thereby contributing to increased pain (Woolf and Ma, 2007). NGF is known to sensitize nociceptors to mechanical stimulation in a number of different tissue types, including bone (Nencini et al., 2017). siRNA-mediated knockdown of Piezo2 prevents NGF-induced sensitization in at least some populations of nociceptors, confirming a functional interaction between Piezo2 and NGF *in vitro* (Prato et al., 2017). Here we have shown that Piezo2 and TrkA are co-expressed in many bone afferent neurons, and that Piezo2 knockdown prevents NGF-induced sensitization of almost all Aδ bone afferent neurons to mechanical stimulation. This demonstrates that Piezo2 knockdown alters the function of NGF sensitive bone afferent neurons *in vivo*, and provides a mechanism that could explain increased mechanical sensitivity in patients with painful bony pathologies.

Several alternatively spliced transcript variants of the Piezo2 gene have been described, but the nature of the proteins they each encode in the rat is not currently known. Interestingly, sensory neurons in humans and mice express at least 16 or 17 different splice variants of the Piezo2 gene, the sequences encoded by these variants are highly conserved from fish through to humans, and combinations of multiple variants can be expressed in peripheral sensory neurons (Szczot et al., 2017). This same study also demonstrated that distinct isoforms of Piezo2 impart different functional properties on different types of sensory neurons (Szczot et al., 2017). Given the reported similarities in the splice variants between multiple species, it seems likely that these findings will also apply to the rat, but this remains to be shown. Whilst our findings suggest that a small isoform may be important for normal function in a population of sensory neurons that innervate bone, we cannot comment on whether it is itself driving changes in mechanically sensitivity in bone afferent neurons, and cannot exclude the possibility that other isoforms, including isoforms not detected by our Western blot analysis, may also be involved. However, based on mapping of the small isoform to the N-terminus of Piezo2, we predict that it does not form part of its pore forming domain, because the pore forming domain of Piezo proteins appear to be confined to the C terminus (Coste et al., 2015), and so is not responsible for permeability of the channel on its own. Future work could be directed at identifying which isoforms are differentially expressed in bone afferent neurons, and to explore the impact these have on their function.

It is interesting that Piezo2 knockdown does not prevent Aδ bone afferent neurons from responding to noxious mechanical stimulation, or contribute to setting their threshold for mechanical activation. Whilst this could be related to incomplete knockdown of Piezo2, it is also possible that other mechano-transducers are expressed by Aδ bone afferent neurons, and that these could be more important than Piezo2 for these functions. The molecular identity of the other mechanosensitive channels that contribute to the responses of high threshold mechanoreceptors to noxious mechanical stimulation are still poorly understood (Delmas et al., 2018).

## Conclusion

Piezo2 contributes to the response of Aδ bone afferent neurons to noxious mechanical stimulation and interactions with NGF are likely to be important for its function. Thus Piezo2 and some of its interaction partners might constitute targets for therapeutic benefit in painful conditions driven by mechanical disturbance in bone.

## Data Availability Statement

The raw data supporting the conclusions of this article will be made available by the authors, without undue reservation.

## Ethics Statement

The animal study was reviewed and approved by the University of Melbourne Animal Experimentation Ethics Committee.

## Author Contributions

JI, SN, MM, and SM were involved in conception and design of the work. JI, SN, MM, AJ, and JT were involved in acquisition, analysis, and interpretation of data for the work. JI, SN, MM, AJ, JT, and SM were involved in drafting the work and revising it critically for important intellectual content. JI and SN approved the final version of the manuscript, agreed to be accountable for all aspects of the work in ensuring that questions related to the accuracy or integrity of any part of the work are appropriately investigated and resolved. All authors contributed to the article and approved the submitted version.

## Conflict of Interest

The authors declare that the research was conducted in the absence of any commercial or financial relationships that could be construed as a potential conflict of interest.

## Footnotes

1 https://blast.ncbi.nlm.nih.gov/Blast.cgi

## References

[B1] Arnoldi C. C. Djurhuus J. C. Heerfordt J. Karle A. (1980). Intraosseous phlebography, intraosseous pressure measurements and 99mTC-polyphosphate scintigraphy in patients with various painful conditions in the hip and knee. *Acta Orthop. Scand.* 51 19–28. 10.3109/17453678008990764 7376840

[B2] Bogen O. Alessandri-Haber N. Chu C. Gear R. W. Levine J. D. (2012). Generation of a pain memory in the primary afferent nociceptor triggered by PKCepsilon activation of CPEB. *J. Neurosci.* 32 2018–2026. 10.1523/jneurosci.5138-11.2012 22323716 PMC3305286

[B3] Bogen O. Joseph E. K. Chen X. Levine J. D. (2008). GDNF hyperalgesia is mediated by PLCgamma, MAPK/ERK, PI3K, CDK5 and Src family kinase signaling and dependent on the IB4-binding protein versican. *Eur. J. Neurosci.* 28 12–19. 10.1111/j.1460-9568.2008.06308.x 18616564 PMC2660608

[B4] Bove S. E. Flatters S. J. Inglis J. J. Mantyh P. W. (2009). New advances in musculoskeletal pain. *Brain Res. Rev.* 60 187–201. 19166876 10.1016/j.brainresrev.2008.12.012 PMC4460838

[B5] Chien L. Y. Cheng J. K. Chu D. Cheng C. F. Tsaur M. L. (2007). Reduced expression of A-type potassium channels in primary sensory neurons induces mechanical hypersensitivity. *J. Neurosci.* 27 9855–9865. 10.1523/jneurosci.0604-07.2007 17855600 PMC6672648

[B6] Coste B. Mathur J. Schmidt M. Earley T. J. Ranade S. Petrus M. J. (2010). Piezo1 and Piezo2 are essential components of distinct mechanically activated cation channels. *Science* 330 55–60. 10.1126/science.1193270 20813920 PMC3062430

[B7] Coste B. Murthy S. E. Mathur J. Schmidt M. Mechioukhi Y. Delmas P. (2015). Piezo1 ion channel pore properties are dictated by C-terminal region. *Nat. Commun.* 6:7223. 10.1038/ncomms8223 PMC4445471 26008989

[B8] Coste B. Xiao B. Santos J. S. Syeda R. Grandl J. Spencer K. S. (2012). Piezo proteins are pore-forming subunits of mechanically activated channels. *Nature* 483 176–181. 10.1038/nature10812 22343900 PMC3297710

[B9] Delmas P. Korogod S. Coste B. (2018). “Noxious Mechanosensation,” in *The Oxford Handbook of the Neurobiology of Pain*, ed. Wood J. N. (Oxford: Oxford University Press).

[B10] Du G. Li L. Zhang X. Liu J. Hao J. Zhu J. (2020). Roles of TRPV4 and piezo channels in stretch-evoked Ca(2+) response in chondrocytes. *Exp. Biol. Med.* 245 180–189. 10.1177/1535370219892601 31791130 PMC7045327

[B11] Dubin A. E. Schmidt M. Mathur J. Petrus M. J. Xiao B. Coste B. (2012). Inflammatory signals enhance piezo2-mediated mechanosensitive currents. *Cell Rep.* 2 511–517. 10.1016/j.celrep.2012.07.014 22921401 PMC3462303

[B12] Eijkelkamp N. Linley J. E. Torres J. M. Bee L. Dickenson A. H. Gringhuis M. (2013). A role for Piezo2 in EPAC1-dependent mechanical allodynia. *Nat. Commun.* 4:1682. 10.1038/ncomms2673 PMC3644070 23575686

[B13] Ferrari L. F. Araldi D. Green P. G. Levine J. D. (2020). Marked sexual dimorphism in neuroendocrine mechanisms for the exacerbation of paclitaxel-induced painful peripheral neuropathy by stress. *Pain* 161 865–874. 10.1097/j.pain.0000000000001798 31917777 PMC7085433

[B14] Ferrari L. F. Bogen O. Green P. Levine J. D. (2015). Contribution of Piezo2 to endothelium-dependent pain. *Mol. Pain* 11:65. 10.1186/s12990-015-0068-4 PMC4619430 26497944

[B15] Florez-Paz D. Bali K. K. Kuner R. Gomis A. (2016). A critical role for Piezo2 channels in the mechanotransduction of mouse proprioceptive neurons. *Sci. Rep.* 6:25923. 10.1038/srep25923 PMC4869095 27184818

[B16] Furusawa S. (1970). A neurophysiological study on the sensibility of the bone marrow. *Nippon Seikeigeka Gakkai Zasshi* 44 365–370. 5466754

[B17] Haegerstam G. A. (2001). Pathophysiology of bone pain: a review. *Acta Orthop. Scand.* 72 308–317. 10.1080/00016470152846682 11480611

[B18] Honore P. Mantyh P. W. (2000). Bone cancer pain: from mechanism to model to therapy. *Pain Med.* 1 303–309. 10.1046/j.1526-4637.2000.00047.x 15101876

[B19] Ikeda R. Gu J. G. (2014). Piezo2 channel conductance and localization domains in Merkel cells of rat whisker hair follicles. *Neurosci. Lett.* 583 210–215. 10.1016/j.neulet.2014.05.055 24911969

[B20] Ikeda R. Cha M. Ling J. Jia Z. Coyle D. Gu J. G. (2014). Merkel cells transduce and encode tactile stimuli to drive Abeta-afferent impulses. *Cell* 157 664–675. 10.1016/j.cell.2014.02.026 24746027 PMC4003503

[B21] Ishida T. Tanaka S. Sekiguchi T. Sugiyama D. Kawamata M. (2016). Spinal nociceptive transmission by mechanical stimulation of bone marrow. *Mol. Pain* 12:1744806916628773. 10.1177/1744806916628773 PMC4994861 27030710

[B22] Ivanusic J. (2009). Size, neurochemistry, and segmental distribution of sensory neurons innervating the rat tibia. *J. Comp. Neurol.* 517 276–283. 10.1002/cne.22160 19757492

[B23] Ivanusic J. J. Mahns D. A. Sahai V. Rowe M. J. (2006). Absence of large-diameter sensory fibres in a nerve to the cat humerus. *J. Anat.* 208 251–255. 10.1111/j.1469-7580.2006.00519.x 16441569 PMC2100196

[B24] Ivanusic J. Mahns D. Sahai V. Vickery R. Rowe M. (2005). An *In Vivo* Electrophysiological preparation for studying the neural mechanisms that mediate bone nociception. *Proc. 35th Annu. Meet. Soc. Neurosci.* 169:3.

[B25] Khasar S. G. Gold M. S. Dastmalchi S. Levine J. D. (1996). Selective attenuation of mu-opioid receptor-mediated effects in rat sensory neurons by intrathecal administration of antisense oligodeoxynucleotides. *Neurosci. Lett.* 218 17–20. 10.1016/0304-3940(96)13111-6 8939470

[B26] Kidd B. L. Photiou A. Inglis J. J. (2004). The role of inflammatory mediators on nociception and pain in arthritis. *Novartis Found Symp.* 260 122–133. 10.1002/0470867639.ch9 15283447

[B27] Lai J. Gold M. S. Kim C. S. Bian D. Ossipov M. H. Hunter J. C. (2002). Inhibition of neuropathic pain by decreased expression of the tetrodotoxin-resistant sodium channel, NaV1.8. *Pain* 95 143–152. 10.1016/s0304-3959(01)00391-8 11790477

[B28] Lemperg R. K. Arnoldi C. C. (1978). The significance of intraosseous pressure in normal and diseased states with special reference to the intraosseous engorgement-pain syndrome. *Clin. Orthop. Relat. Res.* 1978 143–156. 729277

[B29] Li W. W. Sabsovich I. Guo T. Z. Zhao R. Kingery W. S. Clark J. D. (2009). The role of enhanced cutaneous IL-1beta signaling in a rat tibia fracture model of complex regional pain syndrome. *Pain* 144 303–313. 10.1016/j.pain.2009.04.033 19473768 PMC2743308

[B30] Mach D. B. Rogers S. D. Sabino M. C. Luger N. M. Schwei M. J. Pomonis J. D. (2002). Origins of skeletal pain: sensory and sympathetic innervation of the mouse femur. *Neuroscience* 113 155–166. 10.1016/s0306-4522(02)00165-3 12123694

[B31] Mantyh P. W. (2014). The neurobiology of skeletal pain. *Eur. J. Neurosci.* 39 508–519. 10.1111/ejn.12462 24494689 PMC4453827

[B32] Mills C. D. Nguyen T. Tanga F. Y. Zhong C. Gauvin D. M. Mikusa J. (2013). Characterization of nerve growth factor-induced mechanical and thermal hypersensitivity in rats. *Eur. J. Pain* 17 469–479. 10.1002/j.1532-2149.2012.00202.x 22915527

[B33] Morgan M. Nencini S. Thai J. Ivanusic J. J. (2019). TRPV1 activation alters the function of Adelta and C fiber sensory neurons that innervate bone. *Bone* 123 168–175. 10.1016/j.bone.2019.03.040 30936039

[B34] Morgan M. Thai J. Trinh P. Habib M. Effendi K. N. Ivanusic J. J. (2020). ASIC3 inhibition modulates inflammation-induced changes in the activity and sensitivity of Adelta and C fiber sensory neurons that innervate bone. *Mol. Pain* 16:1744806920975950. 10.1177/1744806920975950 PMC7724402 33280501

[B35] Murthy S. E. Loud M. C. Daou I. Marshall K. L. Schwaller F. Kuhnemund J. (2018). The mechanosensitive ion channel Piezo2 mediates sensitivity to mechanical pain in mice. *Sci. Transl. Med.* 10:eaat9897. 10.1126/scitranslmed.aat9897 30305457 PMC6709986

[B36] Narayanan P. Sondermann J. Rouwette T. Karaca S. Urlaub H. Mitkovski M. (2016). Native Piezo2 Interactomics Identifies Pericentrin as a Novel Regulator of Piezo2 in Somatosensory Neurons. *J. Proteome Res.* 15 2676–2687. 10.1021/acs.jproteome.6b00235 27345391

[B37] Nencini S. Ivanusic J. (2017). Mechanically sensitive Adelta nociceptors that innervate bone marrow respond to changes in intra-osseous pressure. *J. Physiol.* 595 4399–4415. 10.1113/jp273877 28295390 PMC5491870

[B38] Nencini S. Ivanusic J. J. (2016). The Physiology of Bone Pain. How Much Do We Really Know? *Front. Physiol.* 7:157. 10.3389/fphys.2016.00157 27199772 PMC4844598

[B39] Nencini S. Ringuet M. Kim D. H. Chen Y. J. Greenhill C. Ivanusic J. J. (2017). Mechanisms of nerve growth factor signaling in bone nociceptors and in an animal model of inflammatory bone pain. *Mol. Pain* 13:1744806917697011. 10.1177/1744806917697011 PMC5407668 28326938

[B40] Nencini S. Ringuet M. Kim D. H. Greenhill C. Ivanusic J. J. (2018). GDNF, Neurturin, and Artemin Activate and Sensitize Bone Afferent Neurons and Contribute to Inflammatory Bone Pain. *J. Neurosci.* 38 4899–4911. 10.1523/jneurosci.0421-18.2018 29712778 PMC6596122

[B41] Nencini S. Thai J. Ivanusic J. J. (2019). Sequestration of artemin reduces inflammation-induced activation and sensitization of bone marrow nociceptors in a rodent model of carrageenan-induced inflammatory bone pain. *Eur. J. Pain* 23 397–409. 10.1002/ejp.1315 30218545

[B42] Portenoy R. K. Payne D. Jacobsen P. (1999). Breakthrough pain: characteristics and impact in patients with cancer pain. *Pain* 81 129–134. 10.1016/s0304-3959(99)00006-8 10353500

[B43] Prato V. Taberner F. J. Hockley J. R. F. Callejo G. Arcourt A. Tazir B. (2017). Functional and Molecular Characterization of Mechanoinsensitive “Silent” Nociceptors. *Cell Rep.* 21 3102–3115. 10.1016/j.celrep.2017.11.066 29241539 PMC5751884

[B44] Qi Y. Andolfi L. Frattini F. Mayer F. Lazzarino M. Hu J. (2015). Membrane stiffening by STOML3 facilitates mechanosensation in sensory neurons. *Nat. Commun.* 6:8512. 10.1038/ncomms9512 PMC4633829 26443885

[B45] Ranade S. S. Woo S. H. Dubin A. E. Moshourab R. A. Wetzel C. Petrus M. (2014). Piezo2 is the major transducer of mechanical forces for touch sensation in mice. *Nature* 516 121–125. 10.1038/nature13980 25471886 PMC4380172

[B46] Sakada S. Taguchi S. (1971). Electrophysiological studies on the free-fiber ending units of the cat mandibular periosteum. *Bull. Tokyo Dent. Coll.* 12 175–197. 5287330

[B47] Seike W. (1976). Electrophysiological and histological studies on the sensibility of the bone marrow nerve terminal. *Yonago Acta Med.* 20 192–211. 1032858

[B48] Singhmar P. Huo X. Eijkelkamp N. Berciano S. R. Baameur F. Mei F. C. (2016). Critical role for Epac1 in inflammatory pain controlled by GRK2-mediated phosphorylation of Epac1. *Proc. Natl. Acad. Sci. U S A.* 113 3036–3041. 10.1073/pnas.1516036113 26929333 PMC4801297

[B49] Starr A. M. Wessely M. A. Albastaki U. Pierre-Jerome C. Kettner N. W. (2008). Bone marrow edema: pathophysiology, differential diagnosis, and imaging. *Acta Radiol.* 49 771–786. 10.1080/02841850802161023 18608031

[B50] Summer G. J. Puntillo K. A. Miaskowski C. Dina O. A. Green P. G. Levine J. D. (2006). TrkA and PKC-epsilon in thermal burn-induced mechanical hyperalgesia in the rat. *J. Pain* 7 884–891. 10.1016/j.jpain.2006.04.009 17157774

[B51] Szczot M. Liljencrantz J. Ghitani N. Barik A. Lam R. Thompson J. H. (2018). PIEZO2 mediates injury-induced tactile pain in mice and humans. *Sci. Transl. Med.* 10:eaat9892. 10.1126/scitranslmed.aat9892 30305456 PMC6875774

[B52] Szczot M. Pogorzala L. A. Solinski H. J. Young L. Yee P. Le Pichon C. E. (2017). Cell-Type-Specific Splicing of Piezo2 Regulates Mechanotransduction. *Cell Rep.* 21 2760–2771. 10.1016/j.celrep.2017.11.035 29212024 PMC5741189

[B53] Urch C. (2004). The pathophysiology of cancer-induced bone pain: current understanding. *Palliat. Med.* 18 267–274. 10.1191/0269216304pm887ra 15198116

[B54] Wang L. Zhou H. Zhang M. Liu W. Deng T. Zhao Q. (2019). Structure and mechanogating of the mammalian tactile channel PIEZO2. *Nature* 573 225–229. 10.1038/s41586-019-1505-8 31435011

[B55] Woo S. H. Lukacs V. De Nooij J. C. Zaytseva D. Criddle C. R. Francisco A. (2015). Piezo2 is the principal mechanotransduction channel for proprioception. *Nat. Neurosci.* 18 1756–1762. 10.1038/nn.4162 26551544 PMC4661126

[B56] Woo S. H. Ranade S. Weyer A. D. Dubin A. E. Baba Y. Qiu Z. (2014). Piezo2 is required for Merkel-cell mechanotransduction. *Nature* 509 622–626. 10.1038/nature13251 24717433 PMC4039622

[B57] Woolf C. J. Ma Q. (2007). Nociceptors–Noxious Stimulus Detectors. *Neuron* 55 353–364. 10.1016/j.neuron.2007.07.016 17678850

[B58] Yang H. Liu C. Zhou R. M. Yao J. Li X. M. Shen Y. (2016a). Piezo2 protein: A novel regulator of tumor angiogenesis and hyperpermeability. *Oncotarget* 7 44630–44643. 10.18632/oncotarget.10134 27329839 PMC5190124

[B59] Yang J. Zhang J. Yang H. Li K. Lei X. Xu C. (2016b). The potential role of Piezo2 in the mediation of visceral sensation. *Neurosci. Lett.* 630 158–163. 10.1016/j.neulet.2016.07.058 27481627

