# Supplementary figures and images for: Piezo2 Knockdown Inhibits Noxious Mechanical Stimulation and NGF-Induced Sensitization in A-Delta Bone Afferent Neurons
Source: Front Physiol. 2021 Jul 15;12:644929. doi: 10.3389/fphys.2021.644929 (PMC8320394; doi:10.3389/fphys.2021.644929)

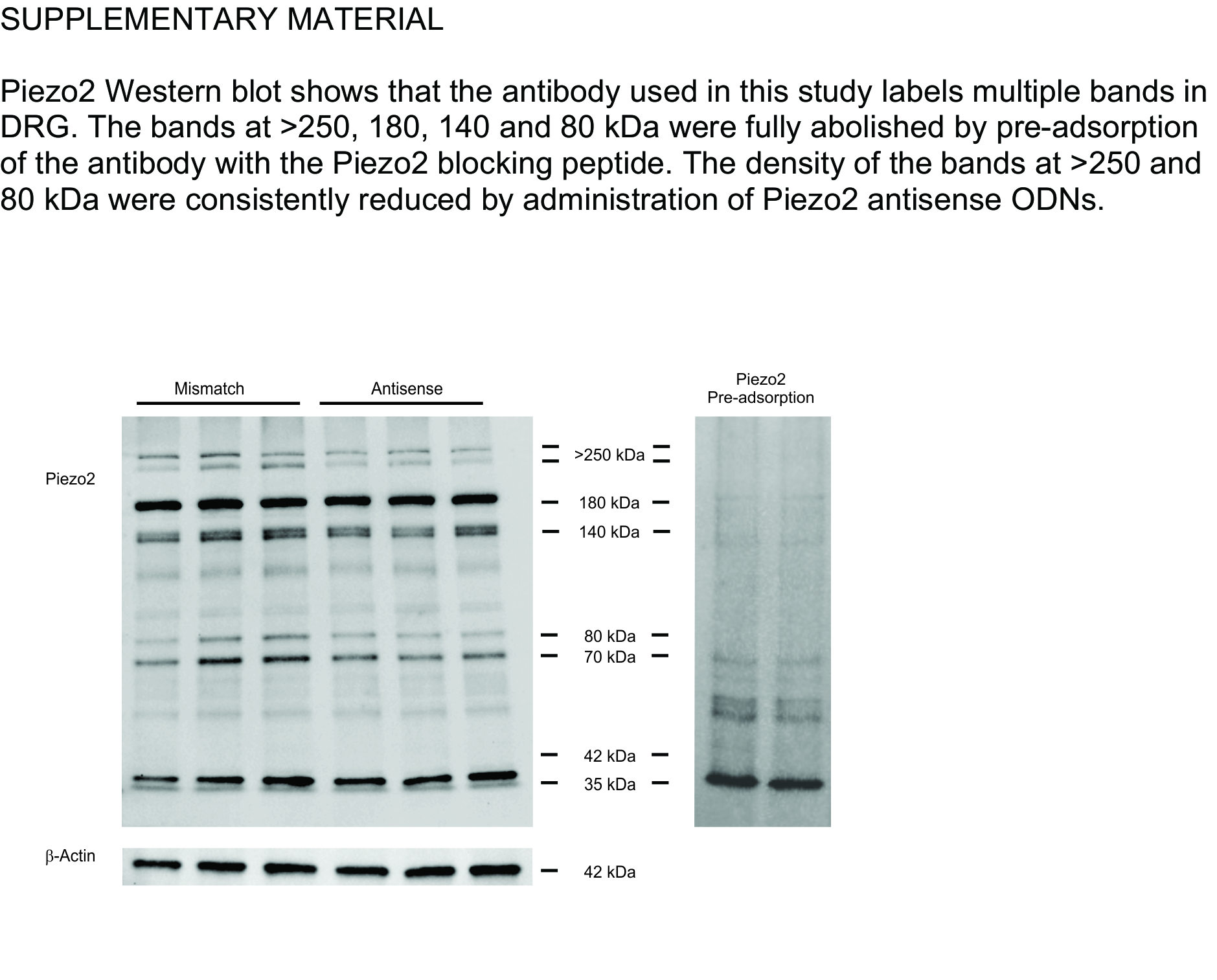

Supplement: Supplementary file 1 [file Image_1.TIF]
